# Use of neuromuscular blockade for neck dissection and association with iatrogenic nerve injury

**DOI:** 10.1186/s12871-023-02217-7

**Published:** 2023-07-28

**Authors:** Joshua D. Smith, Graciela Mentz, Aleda M. Leis, Yuan Yuan, Chaz L. Stucken, Steven B. Chinn, Keith A. Casper, Kelly M. Malloy, Andrew G. Shuman, Scott A. McLean, Andrew J. Rosko, Mark E. P. Prince, Kevin K. Tremper, Matthew E. Spector, Samuel A. Schechtman

**Affiliations:** 1 Department of Otolaryngology – Head and Neck Surgery, Michigan Medicine - University of Michigan, Ann Arbor, MI 48109 USA; 2 Department of Anesthesiology, Michigan Medicine - University of Michigan, 1H247 UH, SPC 5048, 1500 E. Medical Center Dr, Ann Arbor, MI 48109 USA; 3 Rogel Comprehensive Cancer Center, Michigan Medicine University of Michigan, Ann Arbor, MI 48109 USA

**Keywords:** Neck dissection, Lymphadenectomy, Nerve monitoring, Neuromuscular blockade, Nerve, Injury, Iatrogenic

## Abstract

**Background:**

Cranial nerve injury is an uncommon but significant complication of neck dissection. We examined the association between the use of intraoperative neuromuscular blockade and iatrogenic cranial nerve injury during neck dissection.

**Methods:**

This was a single-center, retrospective, electronic health record review. Study inclusion criteria stipulated patients > 18 years who had ≥ 2 neck lymphatic levels dissected for malignancy under general anesthesia with a surgery date between 2008 – 2018. Use of neuromuscular blockade during neck dissection was the primary independent variable. This was defined as any use of rocuronium, cisatracurium, or vecuronium upon anesthesia induction without reversal with sugammadex prior to surgical incision. Univariate tests were used to compare variables between those patients with, and those without, iatrogenic cranial nerve injury. Multivariable logistic regression determined predictors of cranial nerve injury and was performed incorporating Firth’s estimation given low prevalence of the primary outcome.

**Results:**

Our cohort consisted of 925 distinct neck dissections performed in 897 patients. Neuromuscular blockade was used during 285 (30.8%) neck dissections. Fourteen instances (1.5% of surgical cases) of nerve injury were identified. On univariate logistic regression, use of neuromuscular blockade was not associated with iatrogenic cranial nerve injury (OR: 1.73, 95% CI: 0.62 – 4.86, *p* = 0.30). There remained no significant association on multivariable logistic regression controlling for patient age, sex, weight, ASA class, paralytic dose, history of diabetes, stroke, coronary artery disease, carotid atherosclerosis, myocardial infarction, and cardiac arrythmia (OR: 1.87, 95% CI: 0.63 – 5.51, *p* = 0.26).

**Conclusions:**

In this study, use of neuromuscular blockade intraoperatively during neck dissection was not associated with increased rates of iatrogenic cranial nerve injury. While this investigation provides early support for safe use of neuromuscular blockade during neck dissection, future investigation with greater power remains necessary.

**Supplementary Information:**

The online version contains supplementary material available at 10.1186/s12871-023-02217-7.

## Background

Each year in the United States, more than 63,000 individuals will develop mucosal malignancies of the head and neck, accounting for roughly 3% of all cancer diagnoses [1, 2]. Millions more will develop melanoma and non-melanoma skin cancers of head and neck cutaneous subsites [3]. When such cancers are locoregionally advanced, neck dissection at time of primary tumor extirpation is indicated for definitive cancer staging and regional control [4, 5].

Neck dissections are classified according to the extent of lymphatic tissue and non-lymphatic structures dissected [6, 7]. Historically, the radical neck dissection (RND), encompassing resection of all level I-V lymph nodes and sacrifice of the internal jugular vein (IJV), accessory nerve (cranial nerve [CN] XI), and sternocleidomastoid (SCM) muscle, was the accepted standard-of-care [8]. However, the radical neck dissection has now largely been supplanted by the comparatively less morbid but oncologically appropriate modified radical (MRND) and selective neck dissections (SND). The former preserves at least one non-lymphatic structure sacrificed in the RND while the latter preserves all non-lymphatic structures. This change was inspired by high locoregional cure rates with the MRND and SND, improved understanding of lymphatic drainage patterns from specific primary tumor subsites, the efficacy of adjuvant radiation for durable disease control, and a focus on reducing long-term morbidity of cancer treatment [9–11].

Inadvertent injury to cranial nerves can be a devastating, although uncommon, complication of neck dissections. Mechanical injury, thermal injury, or transection of cranial nerves during neck dissection may cause lip asymmetry (marginal mandibular branch, CN VII), vocal cord paralysis and dysphonia (CN X), shoulder dysfunction and pain (CN XI), vocal cord paralysis (CN X), or tongue weakness and dysarthria (CN XII) [12, 13]. While the true incidence of iatrogenic cranial nerve injury is difficult to measure, factors such as surgical expertise, operative volume, and knowledge of anatomic variations in cranial nerve course and branching are critical in mitigating risk [14].

Avoidance of neuromuscular blockade permits intraoperative nerve monitoring (IONM) and direct visualization of neuromuscular activation when dissecting near cranial nerves [15, 16]. The utility and efficacy of this practice in neck dissection is unclear. A recent systematic review identified only three studies on the association of IONM with post-operative shoulder dysfunction after neck dissection, and no conclusions could be drawn [17]. Whether avoidance of neuromuscular blockade during neck dissection reduces risk of iatrogenic cranial nerve injury is unknown. This investigation sought to determine the association between use of neuromuscular blockade and iatrogenic cranial nerve injury in a retrospective cohort of patients with mucosal or cutaneous malignancies of the head and neck undergoing neck dissection.

## Methods

This retrospective cohort study was approved by the University of Michigan Institutional Review Board (HUM00158128) with a waiver of informed consent. This article adheres to the Strengthening the Reporting of Observational Studies in Epidemiology (STROBE) guidelines [18]. The primary objective was to evaluate the association of the use of neuromuscular blockade during neck dissection with iatrogenic cranial nerve injury. We queried the University of Michigan’s perioperative Centricity database (Centricity®; General Electric Healthcare, Waukesha, WI) to identify neck dissection performed under general anesthesia for mucosal or cutaneous malignancies of the head and neck from January 1^st^, 2008 – December 31^st^, 2018, using Current Procedural Terminology (CPT) code 38,724 (“Radical Resection of Lymph Nodes”). Centricity® includes a structured preoperative documentation stored as discrete data elements to permit rapid downstream research data collection and analyses. Each case was manually screened to exclude those patients < 18 years of age, those who had intentional cranial nerve sacrifice due to tumor involvement, those who had only one neck level dissected, and those whose surgeon was not a member of our Otolaryngology—Head and Neck Surgical Oncology division.

From the peri-operative Centricity database, we collected and collated demographic, clinical and peri-operative variables including age, sex, weight, comorbidities, primary cancer site, neck dissection laterality and levels, use of neuromuscular blockade intra-operatively, operative time, and documentation of iatrogenic cranial nerve injury. We defined use of neuromuscular blockade (i.e., patient paralyzed) during surgery as any use of rocuronium, cisatracurium, or vecuronium upon anesthesia induction without reversal with sugammadex prior to surgical incision. In our practice, pre-operative discussion between the anesthesiologist and attending surgeon guides preference for use of neuromuscular blockade on a case-by-case basis. When the attending surgeon prefers no paralytic, our practice is to administer succinylcholine at induction or reverse non-depolarizing neuromuscular blockade just prior to neck dissection. Thus, all other patients in whom sugammadex reversal was administered prior to incision (with use of rocuronium or vecuronium), had use of succinylcholine at induction, or had no paralytic agent administered were considered not to have neuromuscular blockade (not paralyzed) during neck dissection. We routinely perform train-of-four monitoring every 15 min after neuromuscular blockade is administered and/or until 4/4 twitches return. However, given the extent of retrospective review required in train of four monitoring, we elected to define use the definition of neuromuscular blockade consistent with our practice. We defined iatrogenic cranial nerve injury as explicit surgeon documentation in the operative note of mechanical, thermal, or transection injury of the lingual branch of CN V, marginal mandibular branch of CN VII, vagus nerve (CN X), spinal accessory nerve (CN XI), or hypoglossal nerve (CN XII).

### Statistical analysis

Exploratory Data Analysis (EDA) techniques such as histograms, QQ-plots, box plots, scatterplots, and basic descriptive statistics were used to assess the distribution of dependent measures. These were used to identify the distribution of outcomes to determine the appropriate modeling strategies and to explore the most informative transformation of the covariates, confounders and relevant predictors considered. No extreme values were identified, and missing data rates were < 5%, thus complete case analysis was deemed unbiased.

Descriptive statistics were presented as frequencies with percentages, means with standard deviations, or medians with interquartile ranges, as appropriate. Univariate comparisons between those patients with iatrogenic cranial nerve injury versus those without were performed. Standardized differences (SD) between groups are reported, with those variables with SD > 0.2 to be included in subsequent multivariable models. This method allowed us to adjust for potential unbalanced distribution of confounders across groups.

To address our primary study objective, we utilized Chi-square and Point-biserial correlation coefficient tests for binary and continuous variables, respectively. This unadjusted analysis was followed by a multivariable logistic regression model. Assuming a rate of iatrogenic cranial nerve injury of 1.5% in patients not paralyzed versus 3.0% in patients paralyzed during neck dissection, we required a sample size of 3,068 patients to obtain 80% statistical power. This sample size was not practical in our study and would require a multi-institutional effort. Thus, to avoid convergence problems due to the low prevalence of iatrogenic cranial nerve injury in our study, we considered Firth’s estimation. Firth’s Penalized Likelihood is a statistical method used to minimize analytical bias influenced by small samples or rare events [19].

Final variables included in our multivariable logistic regression model were selected following three combined approaches: 1) relevant clinical variables selected a priori by the authors; 2) variables selected based on *p* ≤ 0.05 on univariate comparisons; and 3) variables selected based on SD > 0.2 on univariate comparisons. Prior to model entry, all variables were checked for collinearity using the variance inflation factor (VIF) and a Pearson’s correlation matrix. If a variable pair was determined to be collinear (i.e., either VIF for both variables > 5 or correlation > 0.7), then either the variables were combined into a single concept or the one with a larger univariate effect size was retained in the model. All other variables were considered fit for model entry. Variables selected a priori for model inclusion were age, weight, sex, use of neuromuscular blockade, sugammadex use pre-incision, and an interaction term between neuromuscular blockade and sugammadex use.

The model’s predictive capability was assessed using the area under the receiver operating characteristic curve (AUROC), presented with 95% confidence intervals (CI). Measures of effect size are presented as adjusted odds ratios (OR) with 95% CI. Statistical analysis was performed with SAS v 9.4 (SAS Institute, Cary, NC). A *p*-value ≤ 0.05 was considered statistically significant for all analyses conducted.

## Results

**Fig. 1:**
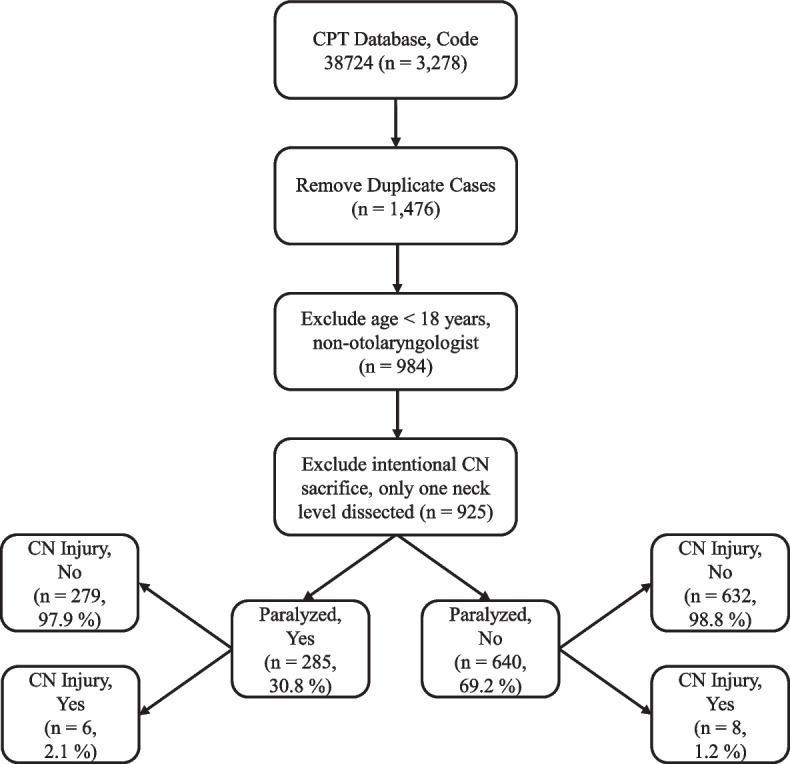
Study flow diagram. N = number of cases. CN; cranial nerve

**Fig. 2:**
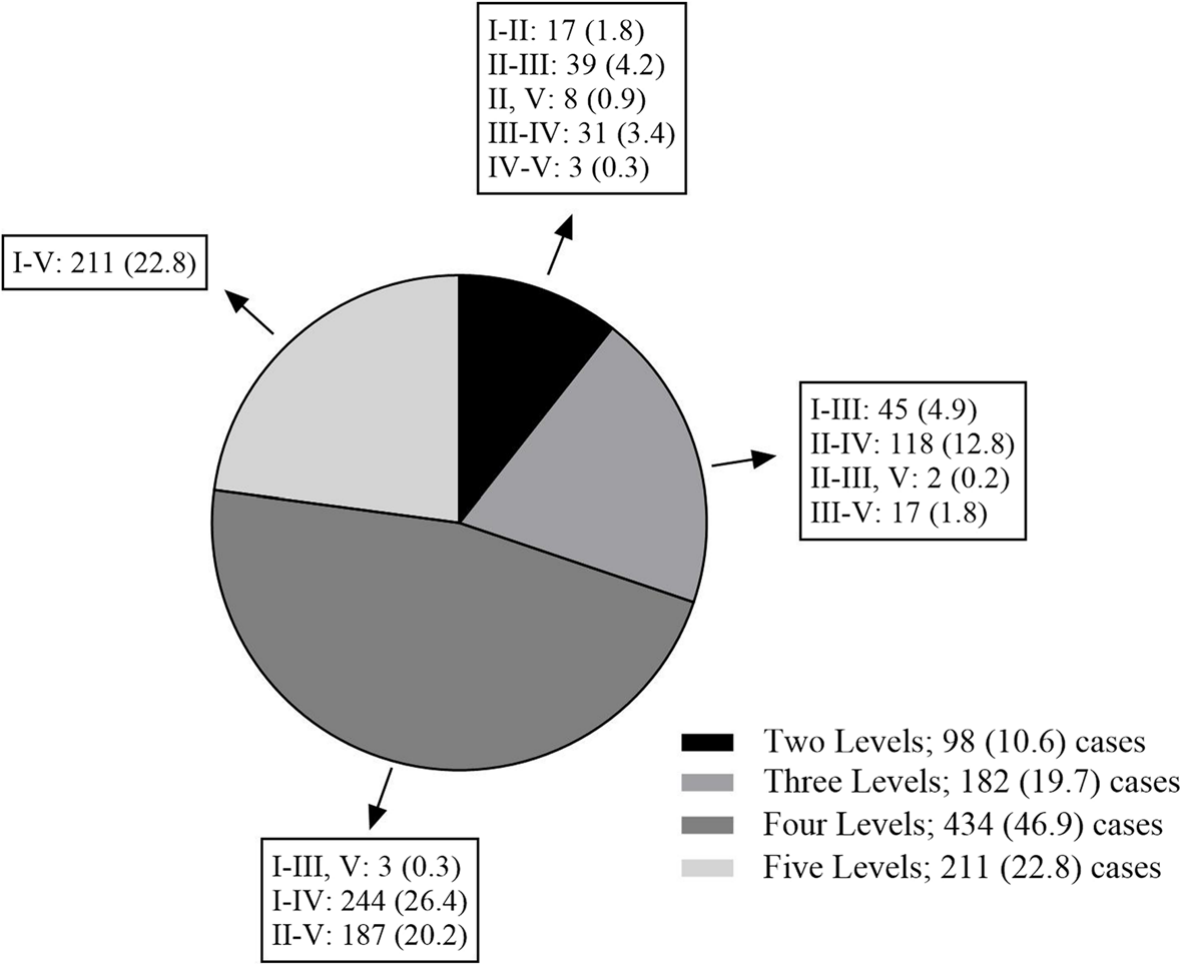
Distribution of neck levels dissected (per side when bilateral neck dissection performed). Data presented as number (%) of total cases

The sample identified 925 distinct surgical cases performed in 897 patients (Fig. 1). Unilateral neck dissections were performed in 862 (93.2%) cases and bilateral neck dissections in 63 (6.8%) cases. The most common operation performed was a SND of levels I-IV followed by a MRND of levels I-V (Fig. 2). Indications for neck dissection in each case are shown in Table 1.

**Table 1:** Indications for neck dissection in each case

	***n* = 925 cases**
Cutaneous Melanoma	315 (34.1)
Cutaneous Non-Melanoma	266 (28.8)
Mucosal SCC^a^	161 (17.4)
Salivary Malignancy^b^	74 (8.0)
Thyroid Malignancy	61 (6.6)
Unknown Primary	18 (1.9)
Other^c^	18 (1.9)
Distant Metastases^d^	12 (1.3)

*SCC* Squamous cell carcinoma

^a^Includes sinonasal, oral cavity, oropharynx, and larynx subsites

^b^Includes major and minor salivary gland subsites

^c^Includes branchial cleft abnormalities, lymphovascular malformations

^d^Includes distant metastases from renal, colon, lung, and breast primaries

A small minority of patients did undergo contralateral neck dissection at a later date than their index operation. Because these were contralateral neck dissections, and thus distinct operative fields, our statistical analysis considered risk of iatrogenic nerve injury per surgical case rather than per patient. This avoided entering the same patient multiple times into the statistical analysis.

Neuromuscular blockade was used during 30.8% of neck dissection cases (*n* = 285 cases). On univariate analysis, there was not an observed difference in demographic characteristics between groups paralyzed versus those not Table 2. Overall, there were 14 iatrogenic cranial nerve injuries documented in both groups (1.5% of surgical cases, 1.4% of neck dissection sides, and 1.6% of patients). The rate of iatrogenic cranial nerve injury did not significantly differ by use of neuromuscular blockade in unadjusted analysis Table 2. The majority of iatrogenic cranial nerve injuries were transection injuries of CN XI Table 3. There was no difference in rate of iatrogenic cranial injury by laterality of neck dissection (unilateral neck dissection, *n* = 13, 1.5% vs. bilateral neck dissection, *n *= 1, 1.5%, *p* = 0.96) or number of neck levels dissected (2–3 neck levels dissected, *n* = 3, 1.1% vs. 4–5 neck levels dissected, n = 11, 1.7%, *p* = 0.47).

**Table 2:** Univariate comparisons in neck dissection cases utilizing neuromuscular blockade vs those not

	**All Cases** **(*n* = 925)**	**Paralyzed** **(*n* = 285)**	**Not Paralyzed** **(*n* = 640)**	**SD**
**Patient Age**	63.8 (15.7)	61.5 (15.7)	64.8 (15.6)	-0.21
**Patient Sex**				0.07
* Female*	275 (29.7)	91 (31.9)	184 (28.8)	
* Male*	650 (70.3)	194 (68.1)	456 (71.2)	
**Patient Weight (kg)**	86.3 (21.6)	84.8 (21.7)	87 (21.5)	-0.10
**Patient ASA Class**				0.14
* 1 or 2*	393 (42.5)	108 (37.9)	285 (44.5)	
* 3 or 4*	532 (57.5)	177 (62.1)	355 (55.5)	
**Patient ASA Class**				0.26
* 1*	20 (2.2)	12 (4.2)	8 (1.2)	
* 2*	373 (40.3)	96 (33.7)	277 (43.3)	
* 3*	518 (56.0)	173 (60.7)	345 (53.9)	
* 4*	14 (1.5)	4 (1.4)	10 (1.6)	
**Operative Time, min**	293 (150)	289 (175)	295 (137)	-0.04
**Iatrogenic Cranial Nerve Injury**	14 (1.5)	6 (2.1)	8 (1.3)	0.07

Data presented as n (%) or mean (standard deviation). *ASA* American Society of Anesthesiologists

**Table 3:** Summary of patients with iatrogenic cranial nerve injury during neck dissection

Patient No	Age	Sex	Levels Dissected	Pathology	Clinical N Stage	Injury Description	Paralyzed
1	73	M	Bi, I-V	Cutaneous SCC	N +	CN XI, transection	Yes
2	62	F	Uni, I-V	Oral cavity SCC	N +	CN XI, avulsion	Yes
3	84	M	Uni, I-V	Larynx SCC	N +	CN XI, transection	Yes
4	56	M	Uni, I-IV	Oropharynx SCC	N +	CN X, transection	Yes
5	62	M	Uni, II-IV	Acinic cell carcinoma, parotid	N +	CN XII, transection	Yes
6	80	F	Uni, I-V	Melanoma	N0	CN XI, transection	Yes
7	51	M	Uni, II-IV	Oropharynx SCC	N +	CN XI, transection	No
8	75	M	Uni, I-V	Melanoma	N0	Marginal mandibular CN VII, transection	No
9	77	M	Uni, II-V	Merkel cell carcinoma	N0	CN XI, transection	No
10	35	M	Uni, I-IV	Oral Cavity SCC	N0	CN XI, transection	No
11	58	F	Uni, II-V	Melanoma	N +	CN XI, transection	No
12	67	M	Uni, I-V	Melanoma	N0	CN XI, transection	No
13	83	M	Uni, II-V	Merkel cell carcinoma	N0	CN XI, transection	No
14	86	M	Uni, II-IV	Cutaneous SCC	N +	CN X, transection	No

*Bi* Bilateral, *Uni* Unilateral, *SCC* Squamous cell carcinoma

Use of neuromuscular blockade during neck dissection was not associated with increased rates of iatrogenic cranial nerve injury (OR: 1.73, 95% CI: 0.62 – 4.86, *p* = 0.30). A summary of variables included in our multivariable logistic regression modeling is provided in Additional file 1. Use of neuromuscular blockade did not reach statistical significance at the α = 0.05 level in our multivariable model (OR: 1.87, 95% CI: 0.63 – 5.51, *p* = 0.26) Table 4.

**Table 4:** Univariate and multivariate logistic regression for predictors of iatrogenic cranial nerve injury during neck dissection

		**Univariate**			**Multivariate**		
	No. Observations	Odds Ratio	95% CI	*p* Value	Odds Ratio	95% CI	*p* Value
**Patient Age**	925	1.016	0.98 – 1.05	0.37	1.004	0.97 – 1.04	0.82
**Patient Sex, Female**	925	0.714	0.21 – 2.39	0.59	1.243	0.41 – 3.78	0.70
**Patient Weight (kg)**	924	1.015	1.00 – 1.04	0.13	1.017	1.00 – 1.04	0.08
**Patient ASA Class**					0.966^*a*^	0.30 – 3.11	0.95
2 vs. 1	925	0.499	0.02 – 10.2	0.65	-	-	-
3 vs. 1	925	0.847	0.05 – 16.0	0.91	-	-	-
4 vs. 1	925	1.414	0.02 – 85.0	0.87	-	-	-
**History, CAD_ICD**^**b**^	924	4.056	1.43 – 11.5	** < *0.01***	2.018	0.49 – 8.29	0.33
**History, CAD_HP**^**c**^	892	3.733	1.22 – 11.4	***0.02***	1.294	0.35 – 4.73	0.70
**History, MI**	893	5.513	1.76 – 17.2	** < *0.01***	2.403	0.66 – 8.76	0.18
**History, Arrhythmia**	892	3.985	1.27 – 12.5	***0.02***	2.829	0.94 – 8.55	0.07
**History, Carotid Atherosclerosi**	507	6.777	1.03 – 44.7	***0.05***	3.351	0.56 – 20.1	0.19
**History, CVA/TIA**	379	3.495	0.14 – 89.0	0.45	3.419	0.15 – 80.3	0.45
**History, Diabetes**	856	0.741	0.13 – 4.13	0.73	0.306	0.05 – 1.93	0.21
**Neuromuscular Blockade Use**	925	1.73	0.62 – 4.86	0.30	1.868	0.63 – 5.51	0.26
**Rocuronium Dose**	186	0.976	0.94 – 1.02	0.23	0.682	0.19 – 2.50	0.56
**Cisatracurium Dose**	20	0.931	0.68 – 1.27	0.65	3.181	0.47 – 21.4	0.23

*CAD* Coronary artery disease, *MI* Myocardial infarction, *CVA/TIA* Cerebrovascular accident/transient ischemic attack

^a^ASA Class 1 or 2 vs. 3 or 4 for multivariate comparison

^b^CAD with implantable cardioverter defibrillator

^c^CAD without implantable cardioverter defibrillator

## Discussion

In our large single-institution cohort, no statistical association between intraoperative use of neuromuscular blockade and iatrogenic cranial nerve injury during neck dissection was found. In our limited sample, this makes a case that the routine use of neuromuscular blockade to optimize perioperative conditions during neck dissection may be safe. Further investigation with greater power remains warranted. This study raises several important points for discussion relevant to anesthetic and surgical disciplines.

Iatrogenic cranial nerve injury during neck dissection can impart long-lasting morbidity adversely impact quality-of-life. For instance, “shoulder syndrome,” caused by injury to the accessory nerve (CN XI), is characterized by chronic shoulder and neck pain, weakness, and restricted range-of-motion [12]. Further, hypoglossal nerve (CN XII) injury may lead to chronic dysarthria, dysphagia, and hemi-tongue atrophy while injury to the vagus nerve (CN X) may cause debilitating dysphagia and dysphonia [20]. Due to a paucity of large, homogeneous studies with precisely defined outcomes, the true prevalence of iatrogenic cranial nerve injury during neck dissection is unknown. While the prevalence of post-operative smile asymmetry (marginal mandibular branch of CN VII) or shoulder syndrome approaches 20% in some series, this likely represents transient neuropraxia and/or additional mechanisms of nerve injury with high rate of spontaneous recovery [21–23]. The rate of objective thermal, mechanical or transection injuries of cranial nerve injuries during neck dissection is likely much lower.

In practice, some head and neck surgeons request that neuromuscular blockade be avoided during neck dissection to monitor muscle activation when dissecting close to cranial nerves. In other head and neck procedures such as thyroid surgery/central neck dissections and parotid surgery, this is a routine practice to allow for IONM of the recurrent laryngeal nerve and facial nerves, respectively [16]. However, the evidence for such practice in lateral neck dissections is scant and limited to case reports of IONM of the accessory nerve, with unproven reduction in post-operative shoulder syndrome [17]. The use of deep neuromuscular blockade has been shown to optimize surgical conditions during laparoscopic surgery and soft tissue operations, including hip arthroplasty, as well as yielding optimal conditions for airway management and endoscopic laryngeal surgery [24–26]. In addition to improved surgical conditions, use of neuromuscular blockade has been demonstrated to result in decreased blood loss in spine surgery [27] and has been demonstrated to reduce oxygen requirements and improve total lung compliance in critically ill patients [28]. Theoretically, use of neuromuscular blockade can also reduce necessity for a deep anesthetic level to prevent movement and muscle response to surgical stimulation in the non-paralyzed patient [29]. This may help improve hemodynamics intraoperatively, which may be vital in patients with cardiovascular disease. On the contrary, other studies have reported an association between neuromuscular blockade and accidental awareness upon induction, maintenance and emergence [30] as well as an increased risk of 30-day readmission [31] and post-operative pneumonia after certain operations [32]. The routine use of neuromuscular blockade monitoring and best practices for antagonism as formulated in the 2023 American Society of Anesthesiologists Practice Guidelines are both vital components of routine anesthetic practice that may reduce associated complications of neuromuscular blockade [33]. Thus, our findings are an important initial step in resolving longstanding controversy in anesthetic management utilizing neuromuscular blockade during neck dissection.

We found no statistically significant association between use of neuromuscular blockade and iatrogenic cranial nerve injury during neck dissection on univariate and multivariate analysis controlling for age, sex, weight, ASA class, paralytic dose and relevant comorbidities. Within our cohort, our statistical methodology supports the conclusion that paralysis was not an independent risk factor for iatrogenic cranial nerve injury.

Importantly, limitations did exist within this investigation. We were unable to assess other crucial variables that may impact risk of iatrogenic cranial nerve injury in this setting, namely anatomic variation of nerve(s), nodal disease burden, and surgical expertise and volume. Further, our surgeons will sometimes request that paralysis be avoided on a case-by-case basis influenced by factors such as nodal disease burden, anatomic variation, and history of radiation. Admittedly, this bias is uncontrolled in our study. Undoubtedly, these factors play a role in risk of iatrogenic cranial nerve injury during neck dissection and should be considered when deciding on use of neuromuscular blockade intraoperatively [34]. We chose to strictly define iatrogenic cranial nerve injury as explicit documentation by the surgeon in the operative report. While this may exclude patients without known cranial nerve injury intraoperatively but with transient weakness noted post-operatively, we feel this strict definition of our primary outcome maximizes external validity of our conclusions. Future prospective investigations may build on our work by performance and documentation of routine post-operative cranial nerve examinations after neck dissection in those patients paralyzed versus those not.

Additionally, we did not assess associated long-term cranial nerve outcomes. Independent of evident nerve transection injuries, we may have missed nerve injuries other than transection that were influenced by intraoperative paralysis leading to long-term symptomatic morbidity. Future studies should evaluate associations between use of neuromuscular blockade and functional outcomes after neck dissection. Further, we did not collect information on previous head and neck surgeries and radiation treatments. Radiation has profound tissue effects including muscle atrophy, soft tissue fibrosis, and vessel wall weakening [35, 36]. As identification and preservation of cranial nerves can be challenging in radiated fields, future studies should evaluate use of neuromuscular blockade during neck dissection in this population specifically. We of course are not able to definitively conclude a causative relationship, or lack thereof, between our variables. A well-designed randomized controlled trial utilizing an even larger population would be valuable [37].

Our utilized thresholds, ASD > 0.2, are higher than those commonly used in prior studies. Of note, all absolute standardized differences are reported in the table for reference, should readers be interested in reviewing characteristics with smaller effect sizes. Cohen [38] suggested that Effect Size Indices of 0.2, 0.5, and 0.8 can be used to represent small, medium, and large effect sizes, respectively. According to Cohen, “a medium effect of 0.5 is visible to the naked eye of a careful observer. A small effect of 0.2 is noticeably smaller than medium but not so small as to be trivial. A large effect of 0.8 is the same distance above the medium as small is below it.” Thus, we chose the value of 0.2 given the sample size of our study.

Additionally, while we considered patients who received rocuronium and vecuronium without sugammadex prior to incision and those who received cisatracurium prior to incision to be paralyzed, there is a possibility that the neuromuscular blockade could have worn off during the neck dissection if not re-dosed. In our practice, if a patient was not to have neuromuscular function intact for nerve monitoring, then providers routinely would use succinylcholine at induction or use rocuronium or vecuronium with sugammadex given before surgical incision. Additionally, there is patient variability in response to neuromuscular blockade which may be influenced by the anesthetic used [39, 40]. Similarly, practice patterns in use of neuromuscular blockade and reversal agents continues to evolve and vary in practice [41, 42].

## Conclusions

The current investigation lends early support that use of neuromuscular blockade in neck dissection does not have a statistically significant association with iatrogenic cranial nerve injury. While anesthetic practice during neck dissection should remain patient-, provider-, and institution-dependent, our data supports that neuromuscular blockade can be considered to optimize anesthetic and surgical conditions in patients undergoing neck dissection. Still, while our data highlights the importance of consideration for this perioperative management strategy, this is a preliminary investigation necessitating further investigation.

## Supplementary Information

**Additional file 1.** 

## Data Availability

The datasets used and/or analysed during the current study are available from the corresponding author on reasonable request.

## Abbreviations

ASA: American Society of Anesthesiologists
AUROC: Area under the receiver operating characteristic curve
Bi: Bilateral
CAD: Coronary artery disease
CI: Confidence interval
CN: Cranial nerve
CPT: Current Procedural Terminology
CVA/TIA: Cerebrovascular accident/transient ischemic attack
EDA: Exploratory data analysis
IJV: Internal jugular vein
IONM: Intraoperative nerve monitoring
Kg: Kilogram
MI: Myocardial infarction
MRND: Modified radical neck dissection
OR: Odds ratio
Q-Q: Quantile–quantile
RND: Radical neck dissection
SCC: Squamous cell carcinoma
SCM: Sternocleidomastoid muscle
SD: Standardized differences
SND: Selective neck dissection
STROBE: Strengthening the Reporting of Observational Studies in Epidemiology
Uni: Unilateral
VIF: Variance inflation factor
