# Supplementary material for: Use of neuromuscular blockade for neck dissection and association with iatrogenic nerve injury
Source: BMC Anesthesiol. 2023 Jul 28;23:254. doi: 10.1186/s12871-023-02217-7 (PMC10375630; doi:10.1186/s12871-023-02217-7)
Supplement: Supplementary file 1 — Additional file 1. [file 12871_2023_2217_MOESM1_ESM.docx]

**ADDITIONAL FILE 1.** Criteria for variable selection in our multivariable logistic regression modeling.

|  |  |
| --- | --- |
| Selected a priori | Patient Sex  Non-Depolarizing Neuromuscular Blockade Use  Patient ASA Class  History, Carotid Atherosclerosis  Patient Weight, kg  History, Diabetes  History, CVA/TIA |
| Univariate comparison, *p* ≤ 0.05 | History, CAD  History, Arrhythmia  History, MI  History, Carotid Atherosclerosis |
| Univariate comparison, SD > 0.2 | Patient Age  Patient ASA Class  Rocuronium Dose  Cisatracurium Dose |
